# Supplementary material for: Eye Lens Organoids Made Simple: Characterization of a New Three-Dimensional Organoid Model for Lens Development and Pathology
Source: Cells. 2023 Oct 18;12(20):2478. doi: 10.3390/cells12202478 (PMC10605248; doi:10.3390/cells12202478)
Supplement: Supplementary file 1 [file cells-12-02478-s001.zip › Supplemental Material.pdf]

Legends to supplemental information

Supplemental Table S1. Gene expression in 2D and 3D cultures, measured by 3' end RNAseq. Columns B-D, gene identification (Ensembl ID, Entrez ID, and gene symbol). Columns E, logFC, log2(expression in 2D cultures / expression in 3D cultures). logCPM, log 2 counts per million. Column G, F, F-test. Column H, PValue, p-value for the expression in 2D and 3D culture, Student's t test. Column I, FDR, adjusted p-value, Benjamini-Hochberg adjustment. Column J, CatMap, a boolean for the gene being present in the CatMap database. Columns K-N, WT_2D_1 to WT_2D_4, gene expression values in 4 2D samples. Columns O-R, WT_3D_1 to WT_3D_4, gene expression values in 4 3D samples. WT_3D_1 to WT_3D_4, gene expression values in 4 3D samples. Columns S-T, logCPM_WT_2D and logCPM_WT_3D, log 2 counts per million in 2D and 3D samples. Column U, gene description.

Supplemental Table S2. Gene expression in laser-microdissected external and internal regions. Columns B-D, gene identification (Ensembl ID, Entrez ID, and gene symbol). Columns E, logFC, log2(expression in internal region / expression in external region). logCPM, log 2 counts per million. Column G, F, F-test. Column H, PValue, p-value for the expression in internal and external regions, Student's t test. Column I, FDR, adjusted p-value, Benjamini-Hochberg adjustment. Column J, CatMap, a boolean for the gene being present in the CatMap database. Columns K-N, µdiss_WT_Inter_1 to µdiss_WT_Inter_4, gene expression values in 4 internal regions. Columns O-Q, µdiss_WT_Exter_1 to µdiss_WT_Exter_3, gene expression values in 3 external regions. Columns R-S, logCPM_µdiss_WT_Inter and logCPM_µdiss_WT_Exter, log 2 counts per million in internal and external regions. Column T, gene description.

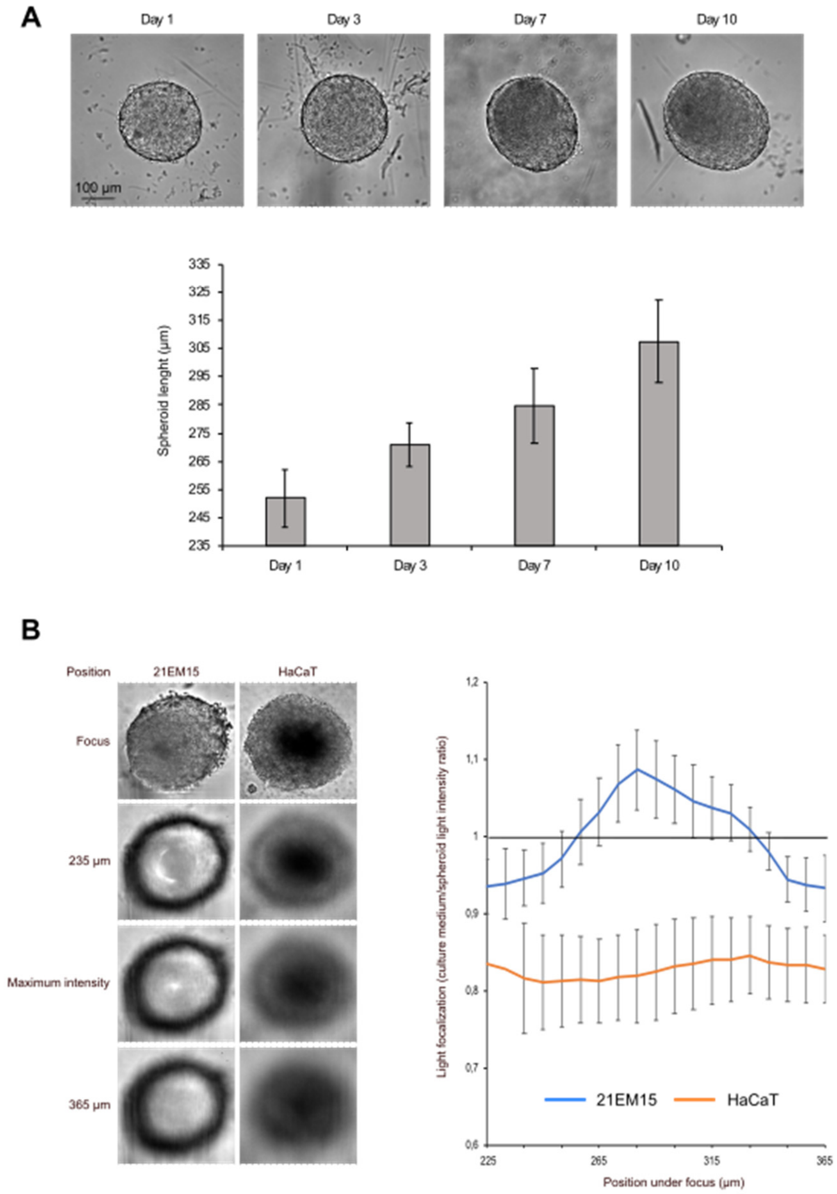

Supplemental Figure S1. Growth pattern and light-focusing properties of 21EM15 spheroid. (**A**) Wide field microscopy images and histogram showing the qualitative and quantitative analysis of the growth of 21EM15 spheroids over time. Graph is representative of three independent experiments with *n*=12 spheroids for each experiment. Error bars represent standard deviations. (**B**) Microscopic

images of 21EM15 and HaCat spheroids. Organoids derived from HaCaT cells are not able to focus light compared to organoids derived from 21EM15 cells. The graph on the right is representative of three independent experiments with *n*=12 spheroids for each experiment. Error bars represent standard deviations.

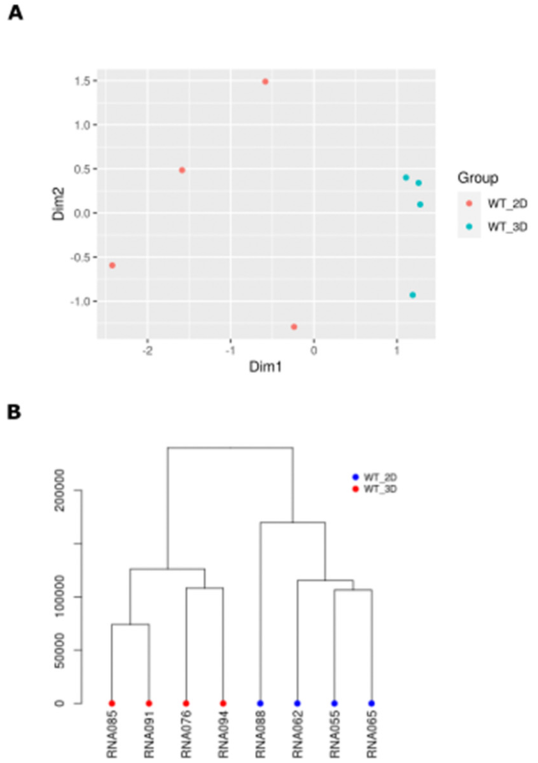

Supplemental Figure S2. Quality control of RNA-seq of 21EM15 3D and 2D cultures. (**A**) Principal Component Analysis (PCA) of RNA-sequencing (RNA-seq)

datasets from lens culture. In the first dimension, the four samples from 3D cultures (blue) are grouped and separated from the four samples from 2D cultures (red). (**B**) Dendrogram based on RNA-seq data. The four 3D samples (spheroids, red) and the 2D samples (blue) segregate into distinct clusters.

A

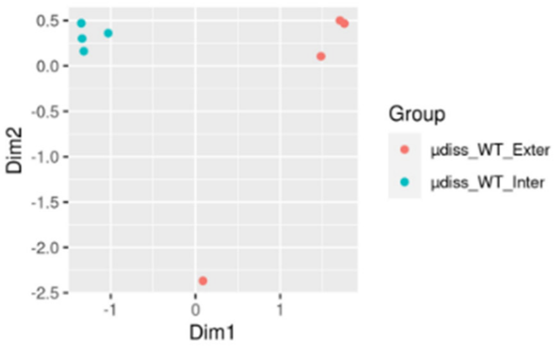

B

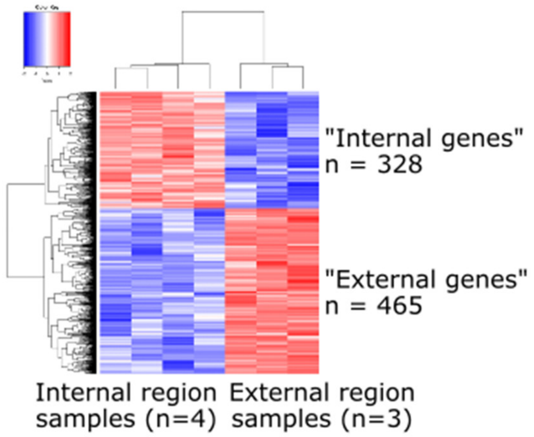

Supplemental Figure S3. Quality control of RNA-seq of laser-captured microdissected 21EM15 spheroids. (**A**) Principal Component Analysis of gene expression. The four samples microdissected from internal regions cluster

together. Three out of four samples microdissected from external regions cluster together, and are clearly separated from the samples microdissected from internal regions in the first dimension. The fourth external region sample differs from the three other ones and we removed it for subsequent analyses. (**B**) Heat map of the 793 DEGs, which separates "internal genes" from "external genes".
